# Impact of Plant–Microbe Interactions with a Focus on Poorly Investigated Urban Ecosystems—A Review

**DOI:** 10.3390/microorganisms12071276

**Published:** 2024-06-23

**Authors:** Pamela Monaco, Apollonia Baldoni, Gino Naclerio, Gabriella Stefania Scippa, Antonio Bucci

**Affiliations:** Department of Biosciences and Territory, University of Molise, Contrada Fonte Lappone, 86090 Pesche, Italy; apollonia.baldoni@unimol.it (A.B.); naclerio@unimol.it (G.N.); scippa@unimol.it (G.S.S.)

**Keywords:** urban green spaces, urbanization, urban rhizomicrobiota, plant–microbe interactions, urban microbial biodiversity

## Abstract

The urbanization process, which began with the Industrial Revolution, has undergone a considerable increase over the past few decades. Urbanization strongly affects ecological processes, often deleteriously, because it is associated with a decrease in green spaces (areas of land covered by vegetation), loss of natural habitats, increased rates of species extinction, a greater prevalence of invasive and exotic species, and anthropogenic pollutant accumulation. In urban environments, green spaces play a key role by providing many ecological benefits and contributing to human psychophysical well-being. It is known that interactions between plants and microorganisms that occur in the rhizosphere are of paramount importance for plant health, soil fertility, and the correct functioning of plant ecosystems. The growing diffusion of DNA sequencing technologies and “omics” analyses has provided increasing information about the composition, structure, and function of the rhizomicrobiota. However, despite the considerable amount of data on rhizosphere communities and their interactions with plants in natural/rural contexts, current knowledge on microbial communities associated with plant roots in urban soils is still very scarce. The present review discusses both plant–microbe dynamics and factors that drive the composition of the rhizomicrobiota in poorly investigated urban settings and the potential use of beneficial microbes as an innovative biological tool to face the challenges that anthropized environments and climate change impose. Unravelling urban biodiversity will contribute to green space management, preservation, and development and, ultimately, to public health and safety.

## 1. Introduction

Over the past few decades, the urbanization process (which began with the Industrial Revolution) has undergone a considerable increase. The percentage of people currently living in cities has nearly doubled since 1950, and it is estimated that the global urbanization level will reach 68% by 2050 [1]. Conspicuous human migration into urban areas led to the formation of increasingly large cities and urban agglomerations, which turned these areas into hot spots of anthropogenic effects on ecosystems [1,2]. Urbanization strongly affects ecological processes (often deleteriously) because it is associated with a decrease in green spaces, loss of natural habitats, increased rates of species extinction, a greater prevalence of invasive and exotic species, and anthropogenic pollutant accumulation [2,3].

Urban green spaces are areas of land covered by vegetation within the urban environment. These spaces include public green spaces (such as flowerbeds, parks, riverbanks, green paths, and other public places with grass, trees, and shrubs) and private areas (gardens intended for private use, green spaces belonging to corporate organizations, and indoor potted plants) [3,4,5].

Plants play a central role in urban ecosystems by providing several ecological benefits. They

- Promote flora and fauna conservation via habitat provision;
- Contribute to environmental protection from soil and slope erosion, torrential floods, landslides, and strong winds;
- Participate in the absorption/removal of pollutants, carbon uptake, oxygen release, air quality improvement, soil drainage, atmospheric cooling, temperature change moderation, and mitigation of extreme weather events [4,6,7].

In addition to being essential for the maintenance of healthy and stable urban ecosystems, several studies highlighted a correlation between urban green areas and human psychophysical well-being. Green spaces improve human wellness via engagement with nature, outdoor recreation, physical activity, enhancement in mental recovery and cognitive abilities, and reductions in stress, anxiety, depression, and violent behavior [6,8,9].

Multiple studies link biodiversity to human health [3,10,11]. Urbanization and “macro-biodiversity” loss seem related to a depletion in microbial diversity, which could negatively affect the microbial communities supporting human health and underpin/reinforce the so-called “biodiversity hypothesis”. This hypothesis suggests a link between biodiversity loss and the rapid increase in non-communicable diseases (NCDs). Lower exposure to biodiversity may cause the human immune system to be poorly trained and over-sensitive to normally harmless agents, such as dust particles and pollen [12,13,14]. Conversely, biodiverse green spaces improve human health by favoring exposure to different beneficial environmental microbes [3]. Soils are among the richest and most abundant reserves of environmental microorganisms and supplement the human microbiota in different ways, including via direct contact, transfer of ambient dust, contact with pets, and household dust [12,15,16,17,18,19]. For example, outdoor environments provide beneficial butyrate-producing endospore-forming bacteria, which are normally found in the human gut. Therefore, exposure to healthy urban soils may favor the natural diversification of the gut microbiota, with a consequent improvement in human health [3,20]. Other interesting results derived from recent research have shown that a biodiversity intervention consisting of the introduction of plant and soil materials into kindergarten yards enhanced immune biomarkers and health-associated commensal microbiota in exposed children, with a simultaneous reduction in the relative abundance of potential pathogenic bacteria [13,21]. Therefore, contact with soil biodiversity may help build immune fitness and promote human health by enhancing immunoregulation, providing key metabolites, and supporting metabolic health via a balanced functional profile of the gut microbiota [3,22].

A great variety of microorganisms belonging to different domains (archaea, bacteria, fungi, oomycetes, and protists) and viruses are involved in plant–microbe interactions [23,24]. Interactions between plants and microorganisms date back to the colonization of plants on Earth. Over millions of years, these complex and dynamic associations led to the formation of a “holobiont”, which is a discrete ecological unit derived from the assemblage of the host and other species living in or around it [25]. Plant-associated microorganisms (collectively known as plant microbiota) are distinguished as epiphytes or endophytes depending on their location on the surface or inside vegetal tissues. Phyllospheric microorganisms live on the leaf surface, and rhizospheric microorganisms grow in the soil surrounding plant roots [26,27]. Associations between plants and microorganisms may be more or less tight and of different types. These associations for transient epiphytic saprophytes are relatively negligible, but the interactions involving endophytes, epiphytic commensals, mutualistic symbiotes, and pathogens are of considerable importance [28,29,30]. Microbes can have a positive impact on plant growth and productivity or cause severe phytopathologies that produce extensive damage to agriculture and serious economic repercussions [24,31,32].

With the availability of increasingly sophisticated investigation techniques, the diffusion of sequencing technologies and “omics” analyses, the development of computational biology, and the enormous progress in microscopy, many aspects of microbial life have been revealed. The use of a polyphasic approach based on a combination of novel culture-independent techniques and traditional methods to study microbial communities provided a broader and deeper understanding of microbial structure, function, and dynamics [27,33,34,35]. Therefore, increasing information on the molecular mechanisms underlying plant–microbe interactions and plant microbiota composition has been acquired. Nevertheless, current knowledge on urban contexts is very scarce. Although the benefits of green spaces within cities are well recognized, the ecosystemic role of “urban soil inhabitants” is often not adequately considered. Notably, microbial communities supporting urban green areas are largely overlooked, although they make important contributions to ecosystem services [3,6,36]. Therefore, the present review focused on plant–microbe dynamics within urban green areas, with special attention to the rhizosphere. We provide an overview of the main types of relationships that occur in the rhizosphere and discuss the role of bacteria and fungi in the promotion of plant health and development. This review pays particular attention to factors that drive and shape the composition of the rhizomicrobiota in urban contexts by examining the few papers that address this topic.

## 2. The Rhizosphere: A Theater of Interactions between Plants and Microorganisms

**Figure 1:**
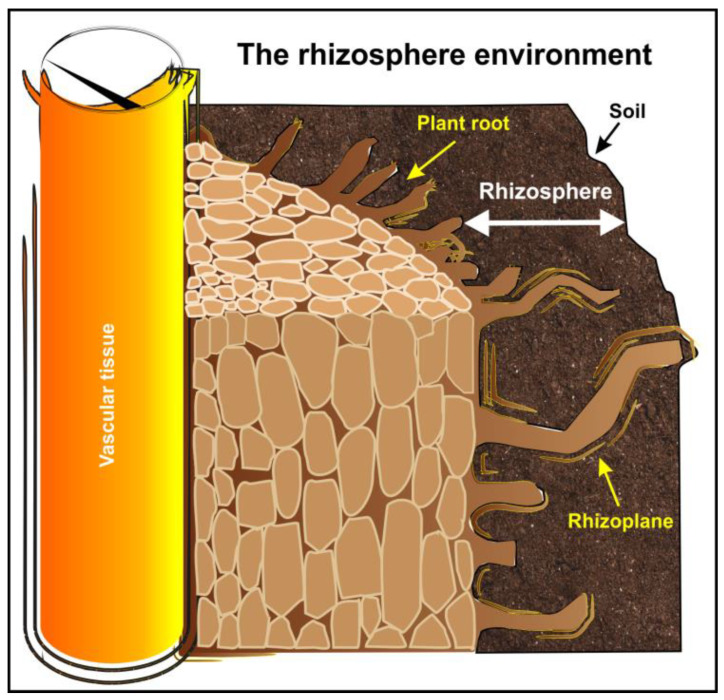
The representation of a root section and the rhizosphere environment.

The German agronomist and plant physiologist Lorenz Hiltner coined the word “rhizosphere” (from the Greek “*rhiza*” = root and “*sphere*” = field or area of influence) in 1904 to indicate the soil compartment influenced by plant roots (Figure 1). He was convinced that root exudates supported the development of diverse microbial communities that significantly affected plant nutrition [26,37,38,39,40,41]. It is common knowledge that the rhizosphere is one of the most complex ecosystems on Earth [42]. It represents a biologically active zone and a hot spot for numerous organisms, such as bacteria, archaea, algae, fungi, oomycetes, protozoa, nematodes, arthropods, and viruses [26,40,43,44,45]. Due to the intrinsic complexity and diversity of plant root systems, the size and shape of the rhizosphere are not defined. The chemical, biological, and physical properties of this environment are not uniform, and these properties vary radially and longitudinally along the root [39].

Exudates released by roots (e.g., sugars, organic acids, amino acids, volatile compounds, phytohormones, and complex mucus-like polymers) are essential nutritional sources that attract and select microorganisms, affecting their density, diversity, and activities [27,40,44]. Root secretions also attract bacteria involved in the formation of biofilms, which are micro-architectural constructions in which bacterial communities are embedded in a self-produced exopolymeric matrix [46]. Bacterial colonization generally occurs at epidermal cell junctions, root hairs, axial groves, cap cells, and lateral root emergence sites [31,39,45]. In addition to the most frequently described bacterial biofilms, mixed biofilms composed of bacterial and fungal species and bacterial biofilms associated with the surface of fungal hyphae appear to be common on plant tissues [31,45].

**Figure 2:**
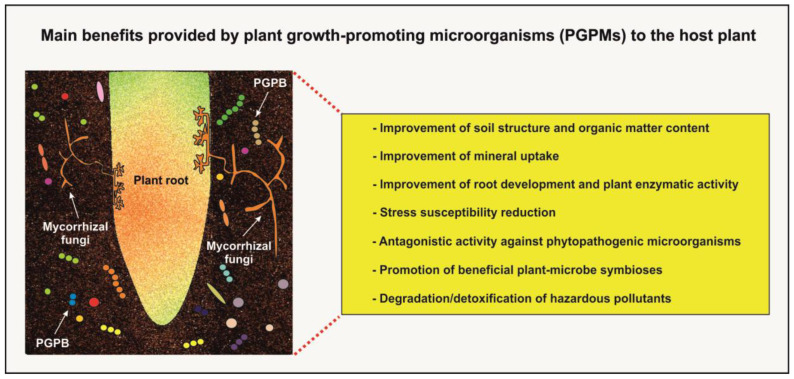
Main beneficial effects exerted by plant growth-promoting microorganisms (PGPMs) on the host plant.

Although many rhizosphere organisms have neutral effects, many others have significant influences on plant growth and health [27,40,44,47]. Pathogenic fungi, oomycetes, bacteria, and nematodes negatively affect the well-being of plants, whereas plant growth-promoting bacteria/rhizobacteria (PGPB/PGPR), nitrogen-fixing bacteria, and mycorrhizal fungi are known for their beneficial effects (Figure 2) [39,44,48,49]. Microorganisms that form biofilms on roots also have a positive impact on plant growth and productivity by conferring protection against abiotic stresses (e.g., salinity, drought, and pollutants) and diseases via the formation of a physical and chemical barrier that prevents pathogen colonization. Rhizosphere microbial biofilms also improve water stability, and they may intervene in organic pollutant and heavy metal biodegradation due to their ability to withstand harsh environmental conditions [31,45,46].

Kloepper and Schroth in 1978 first introduced the expression “plant growth-promoting rhizobacteria” (PGPR) to describe soil bacteria that colonized the roots of the plants after inoculation on seeds and enhanced their growth [39,50]. PGPR, also referred to as plant health-promoting rhizobacteria (PHPR) or nodule-promoting rhizobacteria (NPR), include free-living bacteria, cyanobacteria, plant endophytes, and some bacterial genera (such as *Rhizobium* and *Frankia*) involved in symbiotic relationships. They exert positive effects on plants via numerous mechanisms, as summarized in Table 1. PGPB may act directly (e.g., by facilitating resource acquisition and releasing plant growth-stimulating compounds/phytohormones) or indirectly by exerting antagonistic activity against phytopathogens (biocontrol) and triggering so-called induced systemic resistance [49,51,52,53,54,55,56,57,58,59,60].

Among the direct mechanisms of the rhizobacteria promotion of plant growth, nitrogen fixation is considerably important. Nitrogen is one of the main nutrients for plants and, as it is often present in limited amounts in the soil, botanical species receive enormous benefits from this microbiological process [52,53,61]. The recruitment of nitrogen-fixing bacteria, which, due to the nitrogenase enzymatic complex, reduce unavailable atmospheric nitrogen (N_2_) to assimilable ammonia (NH_3_/NH_4_^+^), allows the plants to satisfy their nutritional requirements by obtaining N directly from the atmosphere [48,62]. Nitrogen can be fixed by PGPB symbiotically or non-symbiotically by free-living diazotrophs, such as *Azospirillum* and *Azotobacter* [48,52]. Several bacterial species in the rhizosphere, such as bacteria in the genera *Bacillus*, *Bradyrhizobium*, *Ensifer*, *Frankia*, and *Rhizobium*, are involved in symbiotic nitrogen fixation processes, which occur within particular root structures called nodules [48,52,63,64,65]. These rhizosphere associations include rhizobia–legume interactions and actinorhizal symbioses. The latter are mutualistic relationships involving actinobacteria of the *Frankia* genus and the roots of non-leguminous plants belonging to eight families of three different orders (*Fagales*, *Rosales*, and *Cucurbitales*) [52,66,67,68]. While *Frankia* exhibits a broad host range, a strong and surprising specificity is observed in the interaction between rhizobia and leguminous plants, which is likely a result of their co-evolution [66,69]. The symbiosis between nitrogen-fixing rhizobacteria and legumes has been studied extensively, which led to broad knowledge of the molecular mechanisms underlying these complex plant–microorganism interactions. Under nitrogen deficiency conditions, leguminous plants release specific flavonoids, such as luteolin, 7,4′-dihydroxyflavone, daidzein, and genistein [70,71]. These compounds attract rhizobia and induce the expression of nodulation genes (nod genes), and the expression of these genes leads to the synthesis of the so-called Nod factors, which are signal molecules (lipochitooligosaccharides) secreted by rhizobia that initiate a cascade of developmental processes in plant roots and result in bacterial invasion and nodule formation [72]. Nitrogen fixation in legumes has considerable ecological, agronomic, and environmental impacts. It is estimated that the biological nitrogen fixation process leads to the release of 52–130 million tons of nitrogen per year globally, with a significant reduction in the use of potentially harmful chemical fertilizers [73]. Due to their unique metabolic capabilities, PGPR are also of great interest in the bioremediation field, where rhizobacterial species able to sequester toxic heavy metals and degrade xenobiotic compounds may be used in association with plants to decontaminate polluted soils [50,51,52,72,74].

Plant roots are also an ideal niche for soil fungi, which live in the rhizosphere as saprotrophs or as mycorrhizal symbionts in association with photosynthetic plants [75,76]. The ability of plants to form mycorrhizas emerged long before (approximately 450 million years ago) they evolved the ability to establish symbiotic associations with rhizobacteria. This may explain why mycorrhizal interactions, unlike rhizobia and their host plants, are ubiquitous throughout the plant domain and relatively non-selective, involving more than 90% of all plant species (angiosperms and gymnosperms) and over 6000 fungal species belonging to the *Glomeromycotina*, *Ascomycotina*, and *Basidiomycotina* subdivisions [77]. Although parasitic and neutral relationships may develop [39], mycorrhizae (from the Greek “*mykos*” = fungus and “*rhiza*” = root) are mostly mutualistic symbioses between soil fungi and plant roots, from which both partners benefit. For their growth and reproduction, colonizing fungi need organic compounds supplied by the host plant. In return, they contribute to improving plant nutrient status by providing minerals and increasing water absorption from the soil and confer resistance to stress and disease [77,78,79,80,81,82]. Mycorrhizal fungi allow efficient horizontal transfer of nutrients linking the roots of several plants (sometimes belonging to different species) via the development of an extensive hyphal network in the soil known as the “wood-wide web” [83,84]. This network of extra-radical hyphae also greatly affects soil quality by promoting soil aggregation and stability via a reduction in erosion and an increase in aeration and water penetration, with a consequent improvement in plant health and productivity [39,85,86]. 

**Figure 3:**
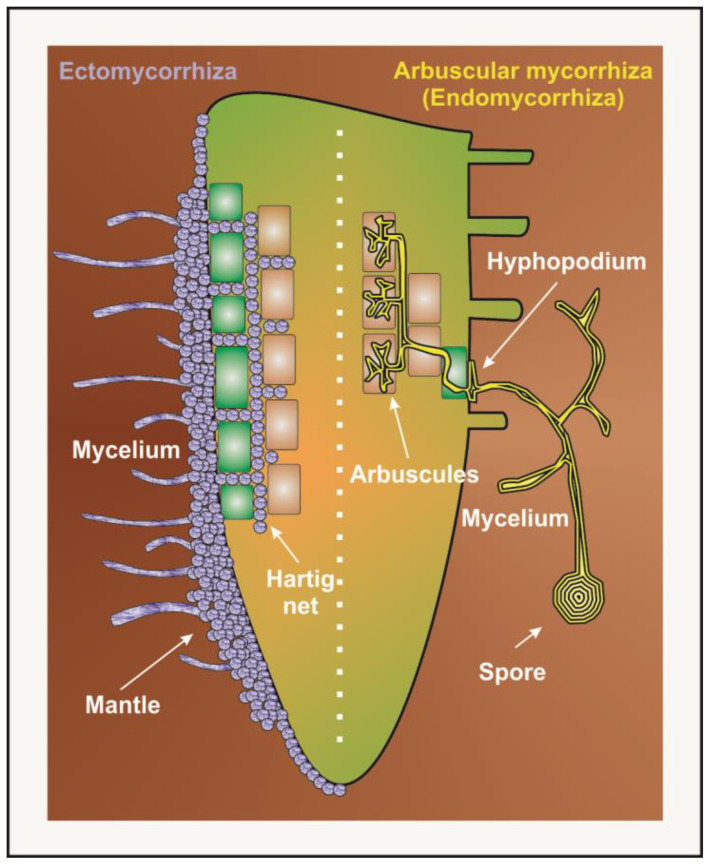
A schematic illustration of the two main types of mycorrhizas: ectomycorrhiza, on the left, and the most widespread endomycorrhiza (arbuscular mycorrhiza), on the right. Green rectangles: root epidermal cells; brown rectangles: root cortical cells.

Mycorrhizal associations are commonly divided into ectomycorrhizal and endomycorrhizal associations, depending on anatomical traits and plant and fungus taxonomic positions (Figure 3) [77].

Ectomycorrhizae (ECMs) include higher plants (trees and shrubs) and fungi almost exclusively belonging to the *Ascomycota* and *Basidiomycota* phyla, whose hyphae never penetrate the lumen of root cells but remain extracellular and induce important changes in root morphogenesis [79]. For ECMs, a dense hyphal covering (fungal sheath or mantle) tightly wraps the root tip, and a network of hyphae, the so-called Hartig net, develops around epidermal and, in some cases, cortical cells, separating them without inducing substantial modifications [75,85,87]. Conversely, fungal hyphae of endomycorrhizae penetrate the cells of the root epidermis and cortex to establish an intracellular symbiosis. Endomycorrhizae are further distinguished into specialized orchid and ericoid mycorrhizae (which are limited to orchids and ericaceous species, respectively) and the most widespread arbuscular mycorrhizas (AMs). In ericoid mycorrhizae, the fungus forms coils that create independent infection units inside root epidermal cells whereas, in orchid mycorrhizae, fungal coils develop primarily in the inner layers of the root [75,76]. Arbuscular mycorrhizae include several plant taxa and fungi of the phylum *Glomeromycota*. The root tips are not generally colonized, and hyphae develop from a spore to produce a specialized swollen structure on the root epidermis known as the hyphopodium [88]. Fungal colonization leads to the formation of so-called arbuscules, which are fan-like, highly branched structures that develop inside inner root cortical cells and represent the main site of nutrient exchange between the two symbiosis partners [75,84,89,90].

In this complex scenario of interactions, rhizosphere microorganisms (particularly bacteria) play a central role because they influence the establishment of mycorrhizal associations and interact with symbiotic fungi during all stages of their life cycle. Microbes are more or less strictly associated with mycorrhizal fungi and colonize the surface of extra-radical hyphae or live as endobacteria in the cytoplasm of AM fungal cells. Physical contact among the partners and the exchange of active molecules (such as strigolactones, Myc factors, chemical volatile compounds, and auxin-like molecules) are the bases of plant–fungus and mycorrhiza–bacterium interactions. Diffusible factors released by rhizosphere bacteria have a negative or positive, in the case of the so-called “mycorrhization helper bacteria” (MHB), impact on the mycorrhization process. Bacteria that establish physical contact with the fungus–root surface may have beneficial effects or possess mycophagic activity [75,87,91].

Therefore, plant–microbe interactions in the rhizosphere are determining factors for plant health and soil fertility and are crucial for the participation of plants in ecosystem functioning. Accordingly, knowledge of microbial diversity and functions is essential for fully understanding the soil–plant interface and ecosystem responses to changing environments [35].

## 3. Rhizosphere Microbial Communities

As previously discussed, the rhizosphere represents an ideal niche for microorganisms and is a site of intense microbial activity. The concentration of microorganisms around plant roots is generally much greater than in bulk soil, on the order of 10^10^–10^12^ cells per gram. The release of low- and high-molecular-weight compounds in the form of exudates in the soil surrounding plant roots (rhizodeposition) results in considerable increases in microbial richness and functional diversity. Therefore, the rhizosphere is an extremely vital and lively environment populated by unique microbial communities and tens of thousands of prokaryotic species [35,47,53].

The structural and functional diversity of rhizosphere microbial communities is influenced by multiple biotic and abiotic factors, such as the climate, season, edaphon, grazing animals, human activities, pesticide treatments, soil type, plant species/cultivars, root exudates, nutrient availability, plant health, and developmental stage [33,92,93,94].

Root-associated communities are different from communities of the non-rhizosphere soil, which suggests that root-associated community establishment is not random but rather driven by host plants that actively shape their rhizomicrobiota. Each plant species selects unique microbial populations, as demonstrated by the fact that diverse plant species grown on the same soil host peculiar communities of microorganisms [27,33,47,92]. The vegetation type strongly affects rhizomicrobiota structure even on a local scale, which was confirmed in a recent study by Liu and colleagues [95]. They analyzed the rhizosphere fungal and bacterial communities of three distinct vegetation types (trees, shrubs, and herbaceous species) and highlighted important differences in microbial diversity. Root exudates are a driving force in attracting and selecting soil microorganisms in this selection process operated by the host plant. Their chemical composition strongly affects rhizosphere community assembly and metabolic potential [27,33,35,96]. Several studies revealed that some exudate components (such as amino acids and short- and long-chain fatty acids) are responsible for the recruitment of specific microbial groups (e.g., *Comamonadaceae*, *Bacillus*, and *Pseudomonas* populations) that are potentially beneficial for plants [27,97,98].

In addition to aspects related to the host plant, data reported in the scientific literature converge in identifying the soil type as another key parameter that greatly influences rhizosphere microbial communities. Rhizomicrobiota composition and diversity are significantly affected by soil organic matter and pH, with lower pH values associated with less diversified communities [33,92,93,99].

Despite the differences related to the plant genotype and environmental factors, similarities in rhizomicrobiota composition and the presence of shared taxa selected from the surrounding bulk soil can be observed for all plants. *Acidobacteria*, *Actinobacteria*, *Bacteroidetes*, *Chloroflexi*, *Firmicutes*, *Proteobacteria*, and *Verrucomicrobia* are the main bacterial phyla generally found in the rhizosphere, whereas *Ascomycota* and *Basidiomycota* are among the dominant fungal groups [92,94,99,100,101,102]. Recent work on an intercontinental scale and based on comparisons between microbial communities of the rhizosphere and bulk soils revealed that some bacterial groups, including *Bacteroidetes* and *Proteobacteria*, were consistently enriched in the rhizosphere, whereas other phyla (such as *Acidobacteria*, *Chloroflexi*, and *Nitrospirae*) were significantly less abundant in rhizosphere soils than in root-free soils [103]. These findings are corroborated by other research, which confirmed the predominance (and/or a significant increase compared to the bulk soil) of bacteria belonging to the *Proteobacteria* phylum in the rhizosphere of different plant species, such as modern wheat cultivars [33], oak trees [100], *Larix decidua* [94], Chinese fir trees [99], and some broad-leaved tree species (*Michelia macclurei*, *Schima superba*, *Phoebe zhennan*, and *Tsoongiodendron odorum*) [101]. The common presence of *Proteobacteria* and *Bacteroidetes* in the rhizosphere of different plants could be explained by the fact that these phyla include bacteria that are copiotrophs (*r*-strategists), which are adapted to carbon-rich conditions, typical of the rhizosphere and required for their high metabolic activity, fast growth, and multiplication [103]. At lower taxonomic levels (e.g., at the genus level), greater differences in the composition of root-associated microbial communities are detected. Some bacterial genera, such as *Rhizobium* and *Mesorhizobium*, are commonly found in the rhizosphere of most plants, but other taxa are enriched only in specific plant groups. For example, an enrichment in *Burkholderia* and *Variovorax* genera was observed in the rhizosphere of *Leguminosae*, whereas an increased abundance of *Pedobacter* and *Aeromicrobium* was detected in *Gramineae* rhizosphere soil [103].

Although it is not easy to draw general conclusions, from the analysis of the few available data on the composition of the rhizosphere microbial communities in urban environments, the presence of recurrent taxa clearly emerged. As well as for the rhizomicrobiota in non-urban contexts, *Proteobacteria* was among the dominant phyla, accompanied by *Acidobacteria* and *Actinobacteria*. Other bacterial groups commonly found were *Bacteroidetes*, *Chloroflexi*, *Firmicutes*, *Gemmatimonadota*, and *Planctomycetes*. For the less studied fungal communities, the *Ascomycota* phylum seemed dominant in the soils of urban parks and showed higher relative abundance values compared to forest soils, followed by fungi belonging to the *Basidiomycota* group [95,104,105].

The rapid development of instrumental and molecular techniques stimulated efforts to decipher the functional features of rhizosphere microbial communities. Because the interface between roots and soil, the rhizosphere hosts abundant and diverse microorganisms that drive carbon and nitrogen dynamics. Recent research based on gene functional prediction analyses demonstrated an enrichment of microbial genes involved in organic compound conversion, carbohydrate metabolism, membrane transport, nitrogen fixation, and denitrification and a great reduction in genes implicated in the nitrification process [94,103]. Compared to bulk soil microbial communities, rhizosphere-inhabiting bacteria generally have greater functional potential for cellulolysis, xylanolysis, chitinolysis, methylotrophy, ureolysis, methanol oxidation, and ligninolysis [103].

## 4. Factors Influencing the Urban Soil (Rhizo)Microbiota

Despite the considerable amount of data on microbial communities and their interactions with plants in natural/rural contexts, current knowledge on rhizosphere microorganisms in urban environments is very scarce. Only a limited number of scientific papers explicitly address the rhizosphere microbial communities of plants located in urban areas [95,104,105]. 

**Figure 4:**
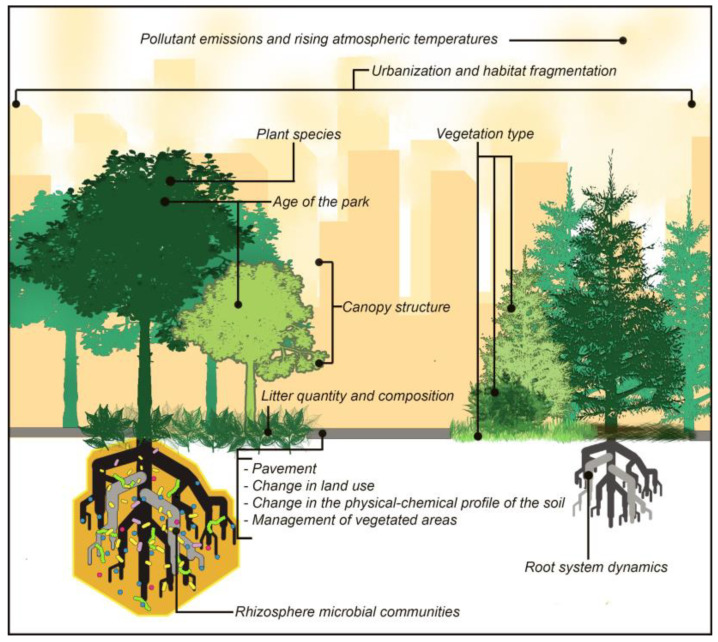
A representation of the main factors influencing urban rhizomicrobiota.

The examination of these works revealed that plants in urban contexts, as in natural environments, exerted an important influence on the rhizosphere microbial communities and affected their structure and composition via the modulation of soil chemistry (Figure 4) [95,104,105,106,107]. For example, plants played a role in modifying the rhizosphere pH via different root-mediated processes. Among them are the release of H^+^ or OH^−^ to compensate for the unbalanced uptake of cations/anions at the soil–root interface; the formation of carbonic acid due to an increase in CO_2_ concentration caused by root exudation and respiration; and redox-coupled reactions by the root system and associated microorganisms. Root-mediated pH changes are considerably important from an ecological perspective, because soil pH is a fundamental parameter that influences the bioavailability of many nutrients and toxic elements, the physiology of plant roots, and, ultimately, the rhizomicrobiota [108,109]. Even forest canopy structure may affect the soil microbiota by controlling the timing, amount, and chemical characteristics of precipitation supplied to the soil [105,110]. The microbiota composition of vegetated soil can vary based on differences in leaf litter quality and quantity, especially for the microbial groups responsible for the decomposition of more recalcitrant (leaf litter) compounds [111].

Differences in microbial α-diversity have been observed in relation to the vegetation type also in urban soils. Greater bacterial diversity was detected in the rhizosphere of herbaceous plants compared to shrubs and trees, as well as in the rhizomicrobiota associated with deciduous trees compared to evergreen trees within urban parks of intermediate age [95,104].

However, if plants exert a preponderant influence on the rhizomicrobiota in natural contexts, the anthropogenic impact becomes the leading factor in shaping the urban soil microbial communities. Human activities such as building and road construction, industrial production, waste management, transport use, and the introduction of new plant and animal species reshape the urban environment and, consequently, the soil as its main component, ultimately affecting the urban soil microbial composition [112]. 

Anthropogenic impact is very complex and includes several physical, chemical, and biological factors, as schematized in Table 2. The “urban heat-island effect” (a non-negligible increase in temperature within cities compared to the surrounding non-urban areas) is among the most relevant physical factors. Such climatic alteration (due to the loss of tree cover, soil sealing that reduces evapotranspiration, and albedo reduction) can lead to the adaptation of certain microbial species to higher temperatures, affecting the overall urban soil microbial community composition. Moreover, the increased tolerance to high temperatures can favor the emergence of new opportunistic pathogens (such as *Candida auris*), with potential implications for human health [112,113]. In addition to the correlation with human health, changes in urban soil biodiversity owed to human disturbance lead to modifications in the structure and functioning of the food web. For example, the increase in opportunistic taxa of bacteria, protists, and nematodes observed in urban ecosystems results in greater nitrogen loss, reduced carbon sequestration, and increased greenhouse gas emissions [3,114,115]. Greater nutrient leaching related to the reduction of ectomycorrhizal fungi in urban environments suggests a decrease in plant growth and health, with repercussions on ecosystem stability [3,116].

Urban soils can exhibit altered water regimes. They are often contaminated with hydrophobic pollutants (such as polycyclic aromatic hydrocarbons, PAHs) and are characterized by increased water repellency. This influences soil microbiota by affecting soil moisture levels and water infiltration rates, leading to water stress in bacteria. On the other hand, due to the widespread soil sealing in metropolitan areas, flooding is also common in urban soils [112]. Flooding can result in anoxic conditions that drive shifts in microbial community composition due to changes in available electron acceptors and altered chemical and physical soil properties. Under flooded conditions, certain bacterial taxa take over while others decrease. For example, it has been reported that representatives of the genus *Aquaspirillum* became predominant in the rhizosphere and rhizoplane of poplar plants grown under these conditions [117].

As previously discussed, a dissimilarity between soil microbial communities associated with distinct tree species was observed in rural forests, which suggests a species-specific effect. Conversely, microbial communities of different plant species seem to converge toward greater similarity when moving toward urban forests due to a greater overlap of shared taxa, not due to a loss of biodiversity [105]. The comparison of the microbiota of urban and rural soils revealed greater microbial diversity within urban green areas exposed to continuous anthropogenic disturbance, rather than in natural contexts, which were characterized by fewer external stressors [104,105]. However, urban environmental conditions, such as drought and extreme temperatures, may act as a filter that favors the survival of tolerant taxa and causes a convergence in the composition of microbial communities residing in the rhizosphere of different plants. This tendency to converge in a similar microbiota composition seemed reinforced at the edges of urban green areas, which are heavily affected by increased solar radiation, wind penetration, and nutrient and pollutant deposition [105]. Several microbial taxa adapted to withstand pollutants, desiccation, and other stresses associated with urbanization have been reported (Table 3) [112]. Among these, bacteria belonging to the *Firmicutes* phylum, which are resistant to extreme environmental changes, showed relatively high percentages in relation to increased anthropic/urban pressures [105,106]. Within *Firmicutes*, aerobic spore-forming bacteria (such as *Bacillus* spp.) play a crucial role in maintaining urban soil health. Indeed, due to their ability to decompose complex organic compounds and fix atmospheric nitrogen, they contribute to carbon and nitrogen biogeochemical cycles. Moreover, *Bacillus* species able to break down PAHs and diesel fuel are involved in the bioremediation of contaminated urban soils [112].

Some plant species are more affected by the impact of anthropogenic factors on their rhizomicrobiota than other species. For example, the *Fagus grandifolia* (beech) rhizomicrobiota showed greater sensitivity to urbanization pressure compared to the *Liriodendron tulipifera* (yellow poplar) microbial community. Similarly, paving seemed to have a greater effect on *Pinus tabuliformis* (pine) root bacterial communities than on the *Fraxinus chinensis* (ash) and *Acer truncatum* (maple) communities [105,106].

The creation of green areas within cities significantly affects the physico-chemical properties of the soil, as well as carbon and nitrogen dynamics and, consequently, the soil microbial community composition. Therefore, the age of a park (i.e., the time since its establishment) may play a role in shaping urban soil microbiota, likely because vegetation in “old parks” has a longer time to modify soil edaphic conditions and the microbial communities therein [104].

In addition to the introduction of “artificial vegetation”, fecal contamination resulting from free-roaming domestic animals, sewage system leaks, or flooding of soil plots with stormwater has a significant biological impact on urban soils [112]. In fact, due to altered particle size distribution and density, urban soils tend to accumulate more fecal bacteria from water sources than the soils of agricultural areas [112,118]. The presence of indicators of fecal contamination and potentially pathogenic bacteria in urban soils could have important repercussions on public health and a significant environmental impact, since fecal bacteria can alter the structure of microbial communities and affect soil health and ecosystem functions.

## 5. Potential of the Rhizomicrobiota in Urban Environments

**Figure 5:**
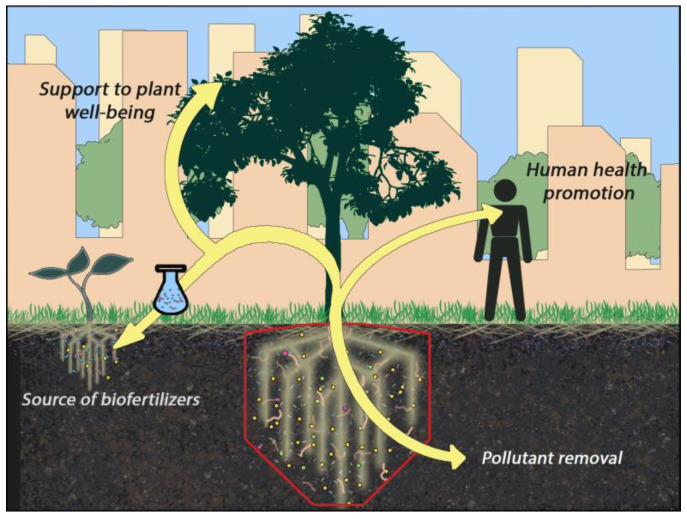
Potential and benefits of rhizosphere microorganisms in urban environments.

Trees in urban environments often exhibit poor growth and reduced survival due to several factors that cause a deterioration of their health status, such as air and soil pollution; nutrient imbalance; increased salinity; water deficit; high limestone (CaCO_3_) levels; soil stripping, mixing, and compaction; high pH values; low moisture retention; and chemical imbalances [119,120]. Rhizosphere microorganisms can increase plant resistance to biotic and abiotic stresses by inducing phenotypic changes in host plants that help the plants cope with the aforementioned unfavorable urban conditions [121]. Vegetal species skillfully use the functional repertoire of rhizosphere microorganisms. Under stress conditions, plants recruit beneficial microbes by secreting a range of chemical factors, following the so-called “cry for help” strategy. Microorganisms may respond by inducing/regulating the expression of plant functional genes related to nutrient uptake and stress resistance via secondary metabolite secretion or volatile compound production. Therefore, rhizosphere microorganisms confer health advantages to plants located in disturbed urban habitats by improving their growth, resistance, and development (Figure 5) [103,122].

In this regard, a valid example is given by polychlorinated biphenyl-degrading microorganisms. Urban environments are often tainted by polychlorinated biphenyls (PCBs), organic compounds with negative/deleterious effects on humans, animals, and ecosystems. Although their production was banned worldwide in 1979, PCBs are persistent pollutants, and highly resistant to degradation, which can contaminate urban soils through historical industrial activities, improper disposal practices of PCB-containing materials, leaks from old equipment, and atmospheric deposition. PCBs can be absorbed by plant roots and translocated to the aerial compartments, causing detrimental effects on plant growth, health, and survival, especially at high concentrations. Since plants are unable to fully mineralize polychlorinated biphenyls, they could adopt the “cry for help” mechanism as an adaptation strategy to mitigate the effects of these highly recalcitrant and toxic pollutants and ensure the holobiont fitness benefiting from microbial associations. In fact, plants growing in contaminated soils modify their root chemistry to recruit, feed, and sustain PCB-degrading microbes in the rhizosphere. In particular, primary metabolites (such as sugars, amino acids, and organic acids) released by plants can be used both as nutrients for the growth of specific microbial taxa and as electron donors to support the microbial aerobic co-metabolism or anaerobic dechlorination of PCBs. Plants can also release secondary metabolites including phenolic molecules like flavonoids, terpenoids, steroids, and alkaloids. These compounds can act as inducers or co-metabolites and trigger the expression of microbial genes encoding for degradative enzymes [123]. Therefore, exposure to PCBs in the soil drives the selection and adaptation of microbial communities with enhanced biodegradative capabilities, leading to complex and cooperative interactions among different bacterial populations and between plants and microbes. For example, bacteria like *Dehalococcoides*, *Dehalogenimonas*, and *Dehalobium* are enriched in PCB-contaminated soils due to their ability to perform anaerobic reductive dechlorination, a key step in PCB degradation. Moreover, it has been reported that *Dehalococcoides mccartyi* often forms consortia with *Methanosarcina* and *Desulfovibrio* populations, which support this species in the dechlorination process. Aerobic bacteria (including *Rhodococcus*, *Pseudomonas*, and *Bordetella*) and fungi are also involved in PCB degradation [123,124], as reported by Sandhu and colleagues [125], who demonstrated the ability of *Rhodococcus* sp. MAPN-1 to improve *Morus alba* (mulberry plant) growth in PCB-spiked soil.

It should be noted that the “cry for help” strategy is not a unidirectional approach used by a plant in stress conditions to maximize its fitness through the services provided by microorganisms. For their part, once established in the rhizosphere after the recruitment and sustenance through root exudates, microbes can manipulate rhizodeposition to ensure and consolidate their metabolic niche [123]. As previously mentioned, high concentrations of PAHs are often detected in urban soils and this can greatly affect and change microbial community composition and activities. However, microorganisms are at the same time the main driver of PAH decomposition. Indeed, many bacterial species (mainly belonging to *Proteobacteria* phylum) break down PAHs and, although only a limited number of them degrade PAHs with five or more aromatic rings, polycyclic aromatic hydrocarbons with high molecular weight can be degraded in a series of processes by consortia of microbes including bacteria and fungi [126]. Considering that exposure to polycyclic aromatic compounds can pose several health risks (carcinogenic and mutagenic changes, respiratory and hematological problems, neurological dysfunctions, etc.), the importance and benefits of PAH-degrading microbes is evident not only from an environmental/ecological perspective, but also for the impact on human health [126,127].

Several industrial manufacturing processes and the use of fertilizers, pesticides, and herbicides in agricultural fields can cause the release of heavy metals into soil and aquatic systems. Heavy metals (e.g., chromium, mercury, lead, arsenic, and cadmium) may persist in soils for a long time and are subject to bioaccumulation. These metals are highly toxic to the environment and have serious effects on plants (productivity reduction and necrosis of plant tissues), animals, human health (causing cancer, skeletal disorders, reproductive and immune system dysfunctions, and cardiovascular diseases), and microbial populations [128,129]. Cadmium (Cd), lead (Pb), mercury (Hg), and arsenic (As) are toxic to many soil microorganisms, inhibiting their growth and metabolic activities, with a consequent reduction in microbial diversity and abundance. For example, *Azotobacter* abundance and nitrogen fixation activity seem to decrease in the presence of elevated concentrations of lead, cadmium, zinc, and copper. A shift towards microbial communities dominated by metal-resistant strains (which have developed mechanisms to tolerate and, sometimes, even utilize heavy metals for their metabolic processes) is often observed in heavy-metal-polluted urban soils. Some bacteria and fungi possess metal-binding proteins and efflux systems to pump excess metals out from their cells [112]. Thus, although heavy metals negatively affect soil microbiota, thanks to their metabolic versatility and ability to transform dangerous compounds into less toxic forms, microorganisms capable of heavy metal detoxification could be employed to remediate polluted urban soils, improving soil health and supporting plant growth [130].

In addition to the removal of pollutants, microorganisms (e.g., bacteria and fungi) may be used as biofertilizers, namely, inocula of beneficial microbes derived from the rhizosphere [119,120,131]. Rapid urbanization, with the consequent decrease in agricultural land area, biodiversity deterioration, climate change, and the excessive use of chemical fertilizers and pesticides in farming practices, have caused significant environmental damage and pose a risk to public health by affecting food security and sustainability in agriculture. Therefore, we must face important challenges in the agricultural field to meet the nutritional requirements of the ever-increasing global population and the growing demand for healthy foods and move toward environmentally friendly practices and sustainable, climate-smart agriculture. Biofertilizers have been designed as valid alternatives to agrochemicals to optimize crop production while preserving environmental health [131,132]. Due to their plant growth-promoting traits, rhizosphere microorganisms play a crucial role in increasing plant biomass and crop yield under greenhouse and field conditions [132]. A recent greenhouse experiment demonstrated that a combination of biochar (pyrolyzed organic waste) and PGPMs as biofertilizers (*Bacillus velezensis* + *Saccharomyces cerevisiae* thermally inactivated) significantly improved growth and nutrient uptake in silver maple (*Acer saccharinum* L.) saplings grown in urban soil [120]. The mycorrhization of *Jacaranda mimosifolia* D. Don with arbuscular fungi promoted the growth of this ornamental plant under abiotic stresses in urban environments by enhancing its ability to acquire minerals [119]. Similarly, *Rhizophagus irregularis* and *Funneliformis mosseae* AMF had strong positive effects on sour orange (*Citrus aurantium* L.) growth [131]. Therefore, microorganisms (particularly rhizosphere microorganisms) represent a valid alternative to chemical fertilizers for improving crop production and plant growth and health while reducing the negative impact of polluting compounds on urban environments, which are generally heavily polluted and subjected to numerous stresses.

## 6. Conclusions

In order to counteract the deleterious effects that rapid and intense urbanization have had on ecological relationships involving humans, microbiota, and the environment, the renewal of urban space is necessary. The “microbiome-inspired green infrastructure” (MIGI) integrative system was recently proposed to promote healthy urban ecosystems via multidisciplinary planning. MIGI is a nature-focused model/approach that aims to counterbalance urban dysbiosis caused by anthropic activities by improving health-promoting interactions between humans and environmental microorganisms while supporting microbe-mediated ecosystem functionality and resilience. The MIGI model involves the introduction of vegetal, animal, and microbial species within urban and inhabited areas to increase the benefits of the ecosystem on the human immune system. Urban parks, green roofs, rain gardens, hedges, wildlife overpasses, and community gardens represent natural reservoirs of microorganisms that produce immunoregulatory molecules and are of considerable importance in urban environments [14,133].

Due to the relevance of the interactions between plants and microbes in the rhizosphere and the potential benefits of rhizosphere microorganisms, further in-depth analyses are required to expand our current knowledge about the composition, function, and dynamics of urban microbial communities and their impact on anthropized environments. The use of microorganisms and the maintenance of thriving microbial populations that provide essential ecosystem services may contribute to urban green space management, preservation, and development and, ultimately, to public health and safety. The incorporation of plant, animal, and microbial species in green infrastructures and landscape architecture and the reconceptualization of interventions from a microbial-focused perspective may help capitalize on the benefits provided by healthy and stable urban ecosystems.

## Figures and Tables

**Table 1:** Overview of some of the main PGPB mechanisms of action and related effects on plants.

PGPB Mechanisms of Action	Direct and Indirect Effects on Plants
Phosphate solubilization; production/release of siderophores and iron sequestration; nitrogen fixation	Improvement in mineral uptake
Production of plant growth-stimulating compounds/regulators and phytohormones (e.g., auxins, cytokinins, gibberellins, and abscisic acid)	Promotion of plant growth and development; adaptive responses to abiotic and biotic stresses
Production of 1-aminocyclopropane-1-carboxylic acid (ACC) deaminase, ACC metabolization	Growth promotion and stress susceptibility reduction
Synthesis of volatile organic compounds (VOCs)	Beneficial effect on growth and development
Enhancement in ERD 15 (Early Responsive to Dehydration 15) gene expression by *Paenibacillus polymyxa*	Increase in drought stress tolerance
Production of antioxidants that degrade ROS (reactive oxygen species)	Improvement in oxidative stress tolerance
Siderophore production; interference with toxin production and quorum sensing systems (resulting in the inhibition of biofilm formation); induced systemic resistance (ISR) activation	Antagonistic activity against phytopathogenic microorganisms (biocontrol)
Production of fungal cell wall degrading enzymes, such as chitinase and β-1,3-glucanase	Antagonistic activity against phytopathogenic fungi

**Table 2:** Factors related to the anthropogenic impact that contribute to shaping soil microbial communities.

Physical factors	Temperature
	Water regime/Soil hydrology changes
	Mixing and disturbance of soil profiles (“Urbopedogenesis”)
Chemical factors	Contamination
	Changes in soil pH
Biological factors	Artificial vegetation
	Contamination

**Table 3:** Examples of bacterial taxa tolerating and thriving in the challenging conditions of urban environments.

*Arthrobacter*	Representatives of *Arthrobacter* genus: - Thrive in high-pH conditions; - Dominate in soils rich in cement dust and construction debris; - Resist desiccation and starvation; - Can degrade hydrocarbons, pesticides, and other pollutants (bioremediation).
*Bacillus*	- Able to break down PAHs and diesel fuel (bioremediation).
*Mycobacterium*	- Highly adaptable and resistant to environmental stressors (e.g., drying and UV radiation); - Certain species can degrade PAHs and other organic pollutants, contributing to the decontamination of polluted urban soils.
*Rhodococcus*	*Rhodococcus* representatives: - Become dominant in urban soils polluted with polychlorobiphenyls and oil; - Capable of degrading a wide range of organic pollutants (bioremediation).
*Streptomyces*	- Often found in soils with high heavy metal levels and altered pH.
